# Synthesis and Anticandidal Activity Evaluation of New Benzimidazole-Thiazole Derivatives

**DOI:** 10.3390/molecules22122051

**Published:** 2017-11-23

**Authors:** Zafer Asım Kaplancıklı, Serkan Levent, Derya Osmaniye, Begüm Nurpelin Sağlık, Ulviye Acar Çevik, Betül Kaya Çavuşoğlu, Yusuf Özkay, Sinem Ilgın

**Affiliations:** 1 Department of Pharmaceutical Chemistry, Faculty of Pharmacy, Anadolu Universty, 26470 Eskisehir, Turkey; serkanlevent@anadolu.edu.tr (S.L.); dosmaniye@anadolu.edu.tr (D.O.); bnsaglik@anadolu.edu.tr (B.N.S.); uacar@anadolu.edu.tr (U.A.C.); betulkaya@anadolu.edu.tr (B.K.C.); yozkay@anadolu.edu.tr (Y.O.); 2 Doping and Narcotic Compounds Analysis Laboratory, Faculty of Pharmacy, Anadolu Universty, 26470 Eskisehir, Turkey; 3 Department of Pharmaceutical Toxicology, Faculty of Pharmacy, Anadolu Universty, 26470 Eskisehir, Turkey; silgin@anadolu.edu.tr

**Keywords:** benzimidazole, thiazole, anticandidal, docking studies, CYP51, cytotoxicity, ergosterol

## Abstract

Azole-based antifungal agents constitute one of the important classes of antifungal drugs. Hence, in the present work, 12 new benzimidazole-thiazole derivatives **3a**–**3l** were synthesized to evaluate their anticandidal activity against *C. albicans*, *C. glabrata*, *C. krusei*, and *C. parapsilopsis*. The structures of the newly synthesized compounds **3a**–**3l** were confirmed by IR, ^1^H-NMR, ^13^C-NMR, and ESI-MS spectroscopic methods. ADME parameters of synthesized compounds **3a**–**3l** were predicted by an in-slico study and it was determined that all synthesized compounds may have a good pharmacokinetic profile. In the anticandidal activity studies, compounds **3c** and **3d** were found to be the most active compounds against all *Candida* species. In addition, cytoxicity studies showed that these compounds are nontoxic with a IC_50_ value higher than 500 µg/mL. The effect of compounds **3c** and **3d** on the ergosterol level of *C. albicans* was determined by an LC-MS-MS method. It was observed that both compounds cause a decrease in the ergosterol level. A molecular docking study including binding modes of **3c** to lanosterol 14α-demethylase (CYP51), a key enzyme in ergosterol biosynthesis, was performed to elucidate the mechanism of the antifungal action. The docking studies revealed that there is a strong interaction between CYP51 and the most active compound **3c**.

## 1. Introduction

Fungal infections represent a serious and presently unresolved health problem, particularly in developed countries. Fungal infections represent 17% of all intensive care unit infections in Europe and the statistics for the United States are similar. Treatment, especially of systemic infections, is accompanied not only by moderate success rates but also by high costs. Furthermore, emerging resistance to commercially offered antifungals has been reported. Additionally, common non-life threatening by aggressive superficial infections such as recurrent vulvovaginal candidiasis, cause important restrictions to patients, resulting in reduced quality of life [1]. Invasive fungal infections and dermatomycoses are the other type of fungal infections, caused by fungal organisms in people with increased vulnerability such as burn patients, neonates, organ transplant patients, and cancer patients receiving chemotherapy. Other risk factors include antibiotic and steroid uses, diabetes, lesions of the dermis and epidermis, neutropenia, malnutrition, and surgery. In recent years, the frequency and severity of fungal infections have increased, mainly in patients with impaired immunity. Besides, an increasing number of fungal cases involved in sepsis is an observed regular trend. Fungal infections in humans can be classified into three groups: (a) infections (mycoses); (b) toxic reactions to toxins present in certain fungi; and (c) allergic reactions to fungal proteins [2,3].

Like their human hosts, fungi are eukaryotic, which limits the number of molecular targets that can be selectively used for drug development without risk of cross-target toxicity. The current portfolio of antifungal compounds approved for the treatment of invasive infections comprises only four classes of antifungal agents active against a limited number of cellular processes. Of perhaps greater concern is the existence of few discovery programs devoted to the development of new antifungal therapeutics because of the probability of limited financial return for the pharmaceutical industry [4].

Presently, azoles, polyenes, pyrimidine analogs, and echinocandins constitute the four classes of antifungal agents used for the treatment of fungal infections [5]. There is also a fifth class, the allylamines, but compounds of this class are only used for treating superficial dermathophytic infections. Current antifungals have many disadvantages such as narrow spectra of activity, toxicity, safety issues, and poor pharmacokinetic properties. In addition, the emergence of strains resistant to the current antifungal agents has highlighted the urgency of developing new drugs with different mechanisms of action that target the biosynthesis of fungal proteins, lipids, and cell walls [6,7,8].

Most antifungal agents, especially the azoles, target the biosynthesis of ergosterol, a main component of the fungal cell wall. This scarcity of fungus-specific targets is a difficult problem because of the frequency of cross-resistance to all drugs with a common target. Resistance to all major current antifungals has been reported in both laboratory and clinic, and continues to be a growing problem in the medical community [9,10]. The growing emergence of azole-resistant yeast and fungal strains is related to the prophylactic use of azole drugs, long treatment programs in the clinic, and usage of agricultural azole fungicides in crop protection. Thus, there is a need for development of new azole antifungal compounds with augmented selectivity for the fungal sterol 14α-demethylase enzyme (CYP51), alternative antifungal approaches, and treatment regimens against drug-resistant strains. Currently, new azole and nonazole CYP51 inhibitors are being developed as the next generation of antifungal drugs [11,12]. The mechanism of action of azole antifungals involves the direct coordination of nucleophilic nitrogen of the azole heterocyclic ring to the sixth ligand of the heme ferric ion, and interactions of the azole drug side chains with the CYP51 polypeptide structure [13,14]. CYP51 is a member of the cytochrome P450 superfamily and is necessary for ergosterol biosynthesis in fungi and cholesterol biosynthesis in mammals. Therefore, fungal CYP51 is the main target for therapeutic azole antifungal agents and agricultural azole fungicides [15]. The similar biosynthesis pathways of ergosterol and cholesterol have led researchers to develop azole inhibitors that are selective for the fungal CYP51 enzyme, and are usually used to treat fungal infections caused by *Candida albicans* and *Aspergillus fumigatus* [16,17].

**Figure 1:**
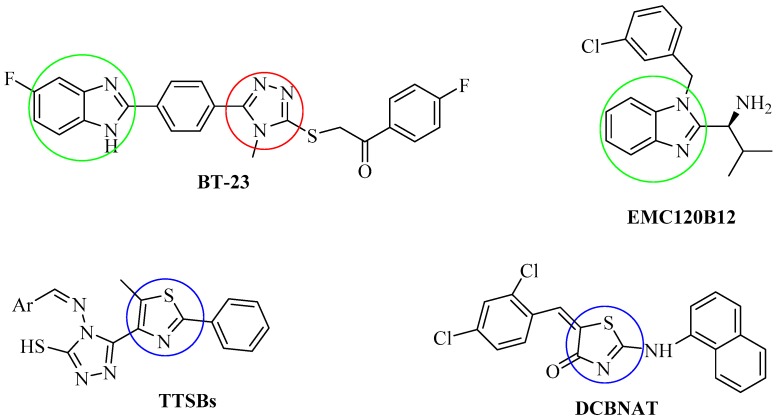
Structures of BT-23, EMC120B12, TTSBs and DCBNAT.

Several triazole-based azole antimycotic agents have been incessantly optimized and presented in clinical practice [18]. However, increased triazole resistance, reproductive toxicity, and hepatic toxicity cases are associated with long-term use of triazole compounds. The capability of triazoles to inhibit CYP-reliant enzymes raises alarms about triazole effects on hormone synthesis and drug metabolism [19,20,21,22]. Therefore, some research group efforts include compound collections of the more encouraging benzimidazole type for antifungal discovery [1,3,14,23,24]. For instance, in our recent study [25], we reported 2-((5-(4-(6-fluoro-1*H*-benzimidazol-2-yl)phenyl)-4-methyl-4*H*-1,2,4-triazol-3-yl)thio)-1-(4-fluorophenyl)ethan-1-one (BT-23, Figure 1), a new benzimidazole-triazole hybrid compound with a significant MIC_50_ (0.78 µg/mL) against *Candida* strains. We also determined that BT-23 acts as an ergosterol biosynthesis inhibitor at 0.78–3.12 µg/mL concentrations.

In addition to classical activity methods, high throughput screening-compatible assays have been adapted in the presence of fungal pathogens to classify novel and selective antifungal lead compounds. In these screenings, a lot of heterocyclic scaffold-based small molecules have been evaluated and several benzimidazole compounds with potential antifungal activity have been identified [1,23,26]. A novel benzimidazole derivative EMC120B12 ((*S*)-2-(1-aminoisobutyl)-1-(3-chlorobenzyl)benzimidazole, Figure 1), displaying high antifungal activities against the major *Candida* species, is an example identified in such screenings [1,26]. 

In addition to benzimidazoles, new thiazolyl-triazole Schiff bases (TTSBs), which have MICs close to those of reference agents ketoconazole and fluconazole, have been reported as potential anticandidal agents [27]. Besides, the newly synthesized thiazolin-4-one derivative, 5-(2,4-dichlorobenzylidene)-2-(naphthalen-1-ylamino)thiazol-4(5*H*)-one (DCBNAT), has been found as highly effective (MIC = 0.015 µg/mL) against a series of pathogenic fungi [28] (Figure 1).

As described above, we have reported the anticandidal potential of benzimidazole-triazole hybrid compounds in our recent work [25]. However, there was no in-silico study, displaying interactions between potent compound (BT-23) and an enzyme like CYP51. Hence, with the purpose of developing new lead antifungal agents, we initially designed novel compounds depending on a similar hybridization strategy as used in our recent work. Benzimidazole and thiazole pharmacophore groups, which have a significance in terms of anticandidal activity, were combined on the same chemical structure and then investigated for their anticandidal effect, cytotoxicity and ability to inhibit CYP51.

## 2. Results and Discussion

### 2.1. Chemistry

**Scheme 1:**
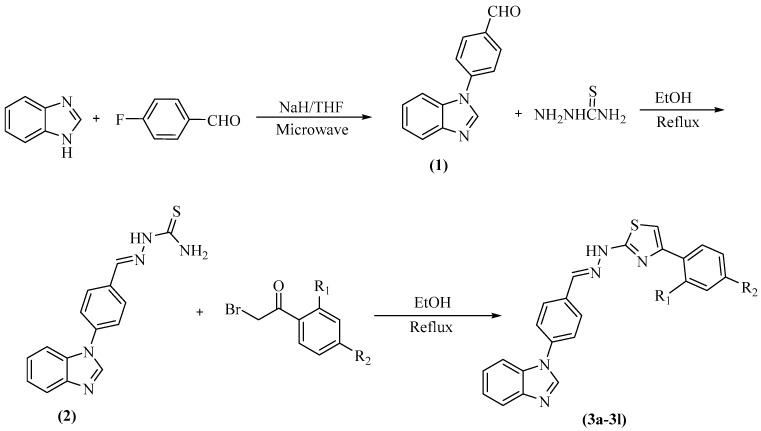
Synthesis of the target compounds **3a**–**3l**.

The compounds **3a**–**3l** were synthesized as outlined in Scheme 1. Firstly, 4-(1*H*-benzimidazol-1-yl)benzaldehyde (**1**) was prepared by reacting 1*H*-benzimidazole and 4-fluorobenzaldehyde under microwave irradiation. Secondly, 4-(1*H*-benzimidazol-1-yl)benzaldehyde (**1**) and hydrazine-carbothioamide were reacted to obtain 2-(4-(1*H*-benzimidazol-1-yl)benzylidene)hydrazine-1-carbothioamide (**2**). Finally, reaction of compound **2** and an appropriate 2-bromoacetophenone afforded the target compounds **3a**–**3l**. In the IR spectra (Supplementary Materials Figures S1 and S5), stretching absorptions at 3097–3218 cm^−1^ indicated the presence of hydrazone group N-H bonds. The stretching absorption at about 1585–1610 cm^−1^ was attributed to C=N double bonds. The stretching absorption belonging to the C–N single bond was determined at 1105–1141 cm^−1^. Stretching absorptions for the out of plane bending of a 1,4-disubstituted benzene moiety was observed at 825–846 cm^−1^. In the ^1^H-NMR spectra (Supplementary Materials Figures S3 and S7), the aromatic protons of benzene, benzimidazole, thiazole and –CH=N– groups were recorded between 6.97 ppm and 9.27 ppm. Methyl protons in compounds **3b** and **3j** were recorded between 2.30 ppm and 2.42 ppm as a singlet. Moreover, the benzimidazole H_2_ and –CH=N– group protons were seen as a singlet between 8.13 ppm and 9.27 ppm. However, the proton of the thiazole ring was recorded as a multiplet (compounds **3a**, **3c**–**3f**, **3h**–**3k**) or a singlet (**3b**, **3g**, **3l**) at 6.19–7.67 ppm. The N–H proton on the hydrazide moiety showed a signal over 12 ppm. In the ^13^C-NMR (Supplementary Materials Figures S4 and S8), all aromatic carbons gave peaks from 102.16 ppm to 168.75 ppm. In fluorinated derivatives (compounds **3f**, **3k** and **3i**), carbon fluorine coupling was observed. In the MS spectra (Supplementary Materials Figures S2 and S6), all masses matched well with the expected [M + H]^+^ or [M + 2H]^2+^ values.

### 2.2. Antifungal Activity Assay

The obtained compounds **3a**–**3l** were evaluated for anticandidal activity against *C. albicans* (ATCC 24433), *C. krusei* (ATCC 6258), *C. parapsilosis* (ATCC 22019) and *C. glabrata* (ATCC 90030) according to the protocol of the EUCAST [29]. The minimum inhibitory concentrations (MICs) of the final compounds were recorded by fluorometric measurements [30,31]. Ketoconazole and fluconazole were used as reference drugs in the activity tests. The antifungal activity results are presented in Table 1.

As regards to the chemical structure of the compounds, the substituents on the phenyl ring are different. According to the antifungal screening results, the most active compound **3c** indicated similar antifungal activity to ketoconazole and fluconazole against all *Candida* strains with a MIC_50_ value of 1.56 µg/mL. Moreover, compound **3d** displayed remarkable activity too. This compound exhibited antifungal activity against *C. glabrata* and *C. krusei* with a MIC_50_ value of 1.56 µg/mL, while it displayed a MIC_50_ value of 3.12 µg/mL against *C. albicans* and *C. parapsilopsis*. The only difference between these two compounds is the substitution at C-4 of the phenyl ring. Compound **3c** bears a nitro group, whereas compound **3d** has a nitrile group at this position. In terms of electronic features, both nitro and nitrile substituents act as strong electron withdrawing groups, so it can be assumed that higher antifungal activity of compounds **3c** and **3d** than other derivatives is related to the presence of electron withdrawing groups, which enhances the antifungal activity.

### 2.3. Quantification of Ergosterol Level

Due to the fact both humans and fungi are eukaryotic, there are comparatively limited antifungal agents able to attack unique fungal targets not shared with human hosts. The fungal cell wall is a however a significant target for selective antifungal drugs owing to the chitin structure that is lacking in the human cells [32,33]. Most of the therapies for fungal infections target the ergosterol biosynthesis pathway, as it is a vital membrane sterol for the normal fungal cell cycle [34].

From this point of view, in the current work newly synthesized benzimidazole-thiazole compounds **3c** and **3d** were selected to investigate their probable mechanism of action. Thus, a LC-MS-MS-based method that quantifies the ergosterol level was applied [25]. Compound **3c**, ketoconazole, and fluconazole were used at 0.78 µg/mL, 1.56 µg/mL, and 3.12 µg/mL concentrations. Ergosterol quantity in negative control samples was regarded as 100%. All concentrations were analyzed in quadruplicate, and the results were expressed as mean ± standard deviation (SD, Table 2).

Ergosterol quantification studies indicated that compounds **3c**, **3d**, and the reference agents significantly reduced the level of ergosterol of *C. albicans* at all tested concentrations. It was determined that tested compounds and reference agent caused a concentration dependent decrease in the ergosterol level. As a result, it can be proposed that compounds **3c** and **3d** have an impact on the biosynthesis pathway of ergosterol.

### 2.4. Cytotoxicity Test

The toxicity of compounds **3c** and **3d** was examined using the MTT assay, which is based upon the reduction of yellow MTT dye by metabolically active eukaryotic and prokaryotic cells to form a purple formazan product. The MTT assay was carried out using healthy NIH/3T3 mouse embryonic fibroblast cell lines (ATCC CRL1658), which are recommended for cytotoxicity screening by ISO (10993-5, 2009) [35]. The IC_50_ values of the compounds are presented in Table 3. The IC_50_ values of compounds **3c** and **3d** were recorded above 500 µg/mL. Consequently, it can be stated that the obtained compounds are nontoxic at their active concentrations against *Candida* species.

### 2.5. Prediction of ADME Parameters

Most new drug candidates fail in clinical trials due to their poor absorption, distribution, metabolism, and excretion (ADME) properties. Late-stage failures cause significant costs in new drug development. The ability to ientiify problematic issues early can significantly decrease the amount of lost time and costs, and rationalize the complete development progression. Therefore, the pharmacokinetic properties of new drug candidates are very vital and should be evaluated as early as possible in the drug development process [36]. Therefore, predictions of ADME parameters of synthesized compounds **3a**–**3l** were performed by using the QikProp 4.8 software [37]. 

This program applies the Lipinski’s rule of five [38] and Jorgensen’s rule of three [39], which evaluate the ADME properties of drug-like compounds, and are important for the optimization of a biologically active compound. The theoretical calculations of ADME parameters (molecular weight, log P, polar surface area (PSA), number of hydrogen donors, number of hydrogen acceptors, number of rotatable bonds and volume) are presented in Table 4, along with the violations of the rules of three and five.

According to Lipinski’s rule of five, all compounds **3a**–**3l** abide by the rules by causing no more than one violation. Furthermore, these compounds fulfil Jorgensen’s rule of three with no more than one violation. Also, it can be seen that all rule of three and five results are within the desired ranges. Thus, it can be stated that all synthesized compounds may have a good pharmacokinetic profile, increasing their pharmacological significance.

### 2.6. Molecular Docking Studies

Docking studies were performed in order to gain more insight into the binding modes of compound **3c** to 14-α-sterol demethylase, which is a key enzyme in ergosterol biosynthesis in fungi. Studies were performed with the X-ray crystal structure of 14-α-sterol demethylase from *Mycobacterium tuberculosis* in complex with our reference agent fluconazole (PDB ID: 1EA1) [40].

According to the antifungal activity results, the compound **3c** shows significant antifungal activity against all *Candida* species with a MIC_50_ value of 1.56 µg/mL. Thus, the main purpose is to investigate the possible interaction of this compound with cytochrome P450 14-α-sterol demethylase from *Candida* species. However, this enzyme is a membrane-bound enzyme and it is difficult to crystallize for X-ray analysis and modelling studies. Moreover, there is no experimental data or crystal structure of this enzyme in Protein Data Bank server. On the other hand, in the database there are two-analogous enzymes from *Candida P450* and *Mycobacterium* P450. These enzymes have high homology and a high degree of similarity between the hydrophobic cavities of the catalytic site [41,42,43,44]. Among them *Mycobacterium* P450 has been resolved with higher resolution. For these reasons, we choose the PDB ID: 1EA1 crystal structure from *Mycobacterium tuberculosis* to obtain a clearer pose.

**Figure 2:**
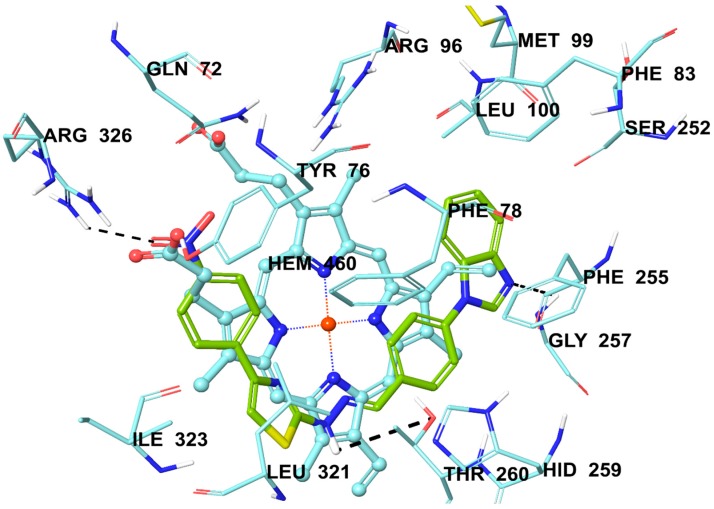
The interacting mode of compound **3c** in the active region of 14 alpha-sterol demethylase. The inhibitor is colored with green and HEM with turquoise.

In docking studies the crystal structure of 14-α-sterol demethylase, which has been reported in a complex with our reference agent fluconazole [40], was used to compare the binding modes of **3c** and fluconazole. It has been reported that the HEM molecule has an interaction with the triazole ring of fluconazole. Two different π-π interactions were also assigned between the 2,4-difluorophenyl moiety and amino acids of Phe83 and Phe255. In Figure 2, the docking pose of the same enzyme reveals that the interactions between compound **3c** and amino acid residues are very important in terms of binding to the active site.

Compound **3c** has some similar interactions to those of fluconazole. The HEM molecule creates a π–π interaction with the phenyl ring, near the benzimidazole ring. It is seen that there are three hydrogen bonds. The nitrogen atom of benzimidazole establishes a hydrogen bond with the amino group of Gly257. The amino of the hydrazone is in an interaction with carbonyl of Thr260. The last hydrogen bond is formed between the nitro group at the C-4 position of the phenyl ring, and the amino group of Arg326. These interactions help explain the stronger anticandidal activity of **3c**. It can be suggested that electron withdrawing groups such as nitro at C-4 of the phenyl ring are very important in terms of binding to the enzyme active site and anticandidal activity. As a result, it is considered that these interactions could explain the better binding capability and stronger activity of compound **3c** than other compounds in the series.

## 3. Materials and Methods

### 3.1. Chemistry

All chemicals were obtained either from Sigma-Aldrich Corp. (St. Louis, MO, USA) or Merck KGaA (Darmstadt, Germany), and used without further chemical purification. Melting points of the compounds were measured by using an automatic melting point determination instrument (MP90, Mettler-Toledo, Columbus, OH, USA) and were presented as uncorrected. ^1^H- and ^13^C-NMR spectra were recorded in DMSO-*d*_6_ by a Bruker digital FT-NMR spectrometer (Bruker Bioscience, Billerica, MA, USA) at 300 MHz and 75 MHz, respectively. The IR spectra of the compounds were recorded using an IRAffinity-1S Fourier transform IR (FTIR) spectrometer (Shimadzu, Tokyo, Japan). MS studies were performed on an LCMS-8040 tandem mass system (Shimadzu, Tokyo, Japan). Chemical purities of the compounds were checked by classical TLC applications performed on silica gel 60 F254 (Merck KGaA). 

#### 3.1.1. Synthesis of 4-(1*H*-Benzimidazol-1-yl)benzaldehyde (**1**)

4-Fluorobenzaldehyde (1.607 mL, 0.015 mol), benzimidazole (1.770 g, 0.015 mol), and sodium hydride (NaH) (0.396 g, 0.016 mol) in DMF (10 mL) were put into a 30 mL microwave synthesis reactor vial (Monowave 300, Anton-Paar, Graz, Austria). The reaction mixture was kept under the conditions of 170 °C and 10 bar for 25 min. After cooling, the mixture was poured into ice-water, the precipitated product was washed with water, dried, and recrystallized from ethanol.

#### 3.1.2. Synthesis of 2-(4-(1*H*-Benzimidazol-1-yl)benzylidene)hydrazine-1-carbothioamide (**2**)

A mixture of 4-(1*H*-benzimidazol-1-yl)benzaldehyde (**1**, 2.887 g, 0.013 mol) and thiosemicarbazide (1.183 g, 0.013 mol) were refluxed in EtOH (50 mL) for 3 h. After completion of the reaction the mixture was cooled in an ice-bath, the precipitated product was filtered, dried, and recrystallized from EtOH.

#### 3.1.3. General Procedure for the Synthesis of the Target Compounds **3a**–**3l**

2-(4-(1*H*-Benzimidazol-1-yl)benzylidene)hydrazine-1-carbothioamide (2, 0.3 g, 0.001 mol) and appropriate 2-bromoacetophenone derivative (0.001 mol) in EtOH (20 mL) were refluxed for 4 h. The mixture was cooled in an ice-bath, the precipitated product was filtered, dried, and recrystallized from EtOH.

*2-(2-(4-(1H-Benzimidazol-1-yl)benzylidene)hydrazineyl)-4-phenylthiazole* (**3a**). Yield: 88%, m.p. = 270–271 °C, FTIR (ATR, cm^−1^): 1610 (C=N), 1141 (C–N), 738, 696. ^1^H-NMR (DMSO-*d*_6_): δ = 7.29–7.36 (3H, m, benzimidazole CH, thiazole CH), 7.42 (2H, t, *J* = 7.20 Hz, phenyl CH), 7.69–7.72 (1H, m, phenyl CH), 7.77 (2H, d, *J* = 8.58 Hz, phenyl CH), 7.80–7.82 (1H, m, benzimidazole CH), 7.88 (2H, d, *J* = 7.17 Hz, phenyl CH), 7.91 (2H, d, *J* = 8.73 Hz, phenyl CH), 8.15 (1H, s, –CH=N–), 8.64 (1H, s, benzimidazole CH), 12.32 (1H, s, –NH). ^13^C-NMR (DMSO-*d*_6_): δ = 104.32, 111.34, 120.41, 123.15, 124.13, 124.34, 126.00, 128.04, 128.19, 129.09, 133.30, 134.21, 135.11, 136.75, 140.49, 143.61, 144.15, 151.11, 168.61. ESI-MS (*m*/*z*): [M + 2H]^2+^: 198 (100%).

*2-(2-(4-(1H-Benzimidazol-1-yl)benzylidene)hydrazineyl)-4-(p-tolyl)thiazole* (**3b**). Yield: 83%, m.p. = 266–267 °C, FTIR (ATR, cm^−1^): 3082 (N–H), 1604 (C=N), 1139 (C–N), 837. ^1^H-NMR (DMSO-*d*_6_): δ = 2.33 (3H, s, –CH_3_), 7.22 (2H, d, *J* = 8.07 Hz, phenyl CH), 7.27 (1H, s, thiazole), 7.33–7.37 (2H, m, benzimidazole CH), 7.68–7.71 (1H, m, benzimidazole CH), 7.75–7.78 (4H, m, phenyl CH), 7.81–7.85 (1H, m, benzimidazole CH), 7.91 (2H, d, *J* = 8.61 Hz, phenyl CH), 8.14 (1H, s, –CH=N–), 8.61 (1H, s, benzimidazole CH), 12.28 (1H, s, –NH). ^13^C-NMR (DMSO-*d*_6_): δ = 21.27, 103.39, 111.30, 120.50, 123.08, 124.07, 124.30, 125.95, 128.17, 129.65, 132.49, 133.34, 134.19, 136.78, 137.30, 140.41, 143.63, 144.37, 151.16, 168.51. ESI-MS (*m*/*z*): [M + 2H]^2+^: 205 (100%).

*2-(2-(4-(1H-Benzimidazol-1-yl)benzylidene)hydrazineyl)-4-(4-nitrophenyl)thiazole* (**3c**). Yield: 89%, m.p. = 274–275 °C, FTIR (ATR, cm^−1^): 3163 (N–H), 1598 (C=N), 1105 (C–N), 846. ^1^H-NMR (DMSO-*d_6_*): δ = 7.49–7.53 (2H, m, benzimidazole CH), 7.76–7.80 (2H, m, benzimidazole CH, thiazole CH), 7.84 (2H, d, *J* = 8.52 Hz, phenyl CH), 7.88–7.91 (1H, m, benzimidazole CH), 7.96 (2H, d, *J* = 8.55 Hz, phenyl), 8.12 (2H, d, *J* = 8.91 Hz, phenyl CH), 8.19 (1H, s, –CH=N–), 8.28 (2H, d, *J* = 8.97 Hz, phenyl CH), 9.27 (1H, s, benzimidazole CH), 12.48 (1H, s, –NH). ^13^C-NMR (DMSO-*d*_6_): δ = 109.30, 112.38, 118.30, 124.15, 124.57, 125.08, 125.60, 126.82, 128.26, 129.21, 132.41, 135.15, 135.60, 140.77, 141.04, 143.02, 146.69, 149.06, 168.97. ESI-MS (*m*/*z*): [M + H]^+^: 441 (100%).

*4-(2-(2-(4-(1H-Benzimidazol-1-yl)benzylidene)hydrazineyl)thiazol-4-yl)benzonitrile* (**3d**). Yield: 85%, m.p. = 283–284 °C, FTIR (ATR, cm^−1^): 3196 (N–H), 1610 (C=N), 1139 (C–N), 837. ^1^H-NMR (DMSO-*d*_6_): δ = 7.33–7.38 (2H, m, benzimidazole CH), 7.67 (1H, s, thiazole CH), 7.68–7.71 (1H, m, benzimidazole CH), 7.77 (2H, d, *J* = 8.58 Hz, phenyl CH), 7.79–7.83 (1H, m, benzimidazole CH), 7.88 (2H, d, *J* = 8.52 Hz, phenyl CH), 7.91 (2H, d, *J* = 8.64 Hz, phenyl CH), 8.06 (2H, d, *J* = 8.43 Hz, phenyl CH), 8.17 (1H, s, –CH=N–), 8.62 (1H, s, benzimidazole CH), 12.39 (1H, s, –NH). ^13^C-NMR (DMSO-*d*_6_): δ = 108.20, 110.10, 111.30, 119.45, 120.50, 123.09, 124.08, 124.28, 125.15, 126.59, 128.27, 133.16, 134.02, 136.90, 139.20, 140.96, 143.62, 144.35, 149.41, 168.95. ESI-MS (*m*/*z*): [M + H]^+^: 421 (100%).

*2-(2-(4-(1H-Benzimidazol-1-yl)benzylidene)hydrazineyl)-4-(4-methoxyphenyl)thiazole* (**3e**). Yield: 81%, m.p. = 253–254 °C, FTIR (ATR, cm^-1^): 3105 (N–H), 1600 (C=N), 1138 (C–N), 831. ^1^H-NMR (DMSO-*d_6_*): δ = 3.77 (3H, s, –OCH_3_), 6.97 (2H, d, *J* = 8.91 Hz, phenyl CH), 7.17 (1H, s, thiazole CH) 7.32–7.37 (2H, m, benzimidazole CH), 7.67–7.71 (1H, m, benzimidazole CH), 7.76 (2H, d, *J* = 8.61 Hz, phenyl CH), 7.77–7.78 (1H, m, benzimidazole CH), 7.80 (2H, d, *J* = 8.85 Hz, phenyl CH), 7.90 (2H, d, *J* = 8.64 Hz, phenyl CH), 8.13 (1H, s, –CH=N–), 8.61 (1H, s, benzimidazole CH), 12.27 (1H, s, –NH). ^13^C-NMR (DMSO-*d_6_*): δ = 55.61, 102.16, 111.31, 114.45, 120.48, 123.09, 124.09, 124.31, 127.33, 128.00, 128.16, 133.33, 134.22, 136.75, 140.36, 143.62, 144.31, 150.87, 159.29, 168.50. ESI-MS (*m*/*z*): [M + 2H]^2+^: 213 (100%)*.*

*2-(2-(4-(1H-Benzimidazol-1-yl)benzylidene)hydrazineyl)-4-(4-fluorophenyl)thiazole* (**3f**). Yield: 80%, m.p. = 260–261 °C, FTIR (ATR, cm^−1^): 3091 (N-H), 1606 (C=N), 1138 (C–N), 825. ^1^H-NMR (DMSO-*d*_6_): δ = 7.24 (2H, t, *J* = 8.91 Hz, phenyl CH), 7.32–7.37 (3H, m, benzimidazole CH, thiazole CH), 7.68–7.71 (1H, m, benzimidazole CH), 7.75-7.81 (3H, m, benzimidazole CH, phenyl CH), 7.88–7.93 (4H, m, phenyl CH), 8.14 (1H, s, –CH=N–), 8.61 (1H, s, benzimidazole CH), 12.30 (1H, s, –NH). ^13^C-NMR (DMSO-*d_6_*): δ = 104.12, 111.30, 115.92 (*J*_2_ = 21.41 Hz), 120.50, 123.08, 124.08, 124.31, 127.98 (*J*_3_ = 8.06 Hz), 128.21, 131.77 (*J*_4_ = 2.55 Hz), 133.33, 134.13, 136.83, 140.58, 143.64, 144.37, 150.14, 162.09 (*J*_1_ = 242.71 Hz), 168.70. ESI-MS (*m*/*z*): [M + 2H]^2+^: 215 (100%).

*2-(2-(4-(1H-Benzimidazol-1-yl)benzylidene)hydrazineyl)-4-(4-chlorophenyl)thiazole* (**3g**). Yield: 84%, m.p. = 279–280 °C, FTIR (ATR, cm^−1^): 3213 (N–H), 1585 (C=N), 1141 (C–N), 831. ^1^H-NMR (DMSO-*d*_6_): δ = 7.33–7.38 (2H, m, benzimidazole CH), 7.43 (1H, s, thiazole CH), 7.48 (2H, d, *J* = 8.58 Hz, phenyl CH), 7.68–7.71 (1H, m, benzimidazole CH), 7.77 (2H, d, *J* = 8.67 Hz, phenyl CH), 7.80–7.82 (1H, m, benzimidazole CH), 7.88–7.93 (4H, m, phenyl CH), 8.15 (1H, s, –CH=N–), 8.64 (1H, s, benzimidazole CH), 12.33 (1H, s, –NH). ^13^C-NMR (DMSO-*d*_6_): δ = 105.16, 111.35, 120.40, 123.17, 124.15, 124.34, 127.71, 128.22, 129.11, 132.43, 133.29, 133.98, 134.15, 136.79, 140.68, 143.61, 144.08, 149.87, 168.75. ESI-MS (*m*/*z*): [M + 2H]^2+^: 207 (100%).

*2-(2-(4-(1H-benzimidazol-1-yl)benzylidene)hydrazineyl)-4-(4-bromophenyl)thiazole* (**3h**). Yield: 85%, m.p. = 274–275 °C, FTIR (ATR, cm^−1^): 3188 (N–H), 1608 (C=N), 1141 (C–N), 829. ^1^H-NMR (DMSO-*d*_6_): δ = 7.33–7.37 (2H, m, benzimidazole CH), 7.44 (1H, s, thiazole CH), 7.61 (2H, d, *J* = 8.55 Hz, phenyl CH), 7.68–7.71 (1H, m, benzimidazole CH), 7.76–7.79 (3H, m, benzimidazole CH, Phenyl CH), 7.83 (2H, d, *J* = 8.49 Hz, phenyl CH), 7.91 (2H, d, *J* = 8.55 Hz, phenyl CH), 8.15 (1H, s, –CH=N–), 8.62 (1H, s, benzimidazole CH), 12.32 (1H, s, –NH). ^13^C-NMR (DMSO-*d*_6_): δ = 105.25, 111.31, 120.49, 121.02, 123.10, 124.09, 124.32, 128.02, 128.23, 132.02, 133.32, 134.10, 134.32, 136.84, 140.69, 143.63, 144.33, 153.18, 168.75. ESI-MS (*m*/*z*): [M + 2H]^2+^: 238 (100%).

*2-(2-(4-(1H-benzimidazol-1-yl)benzylidene)hydrazineyl)-4-(4 (trifluoromethyl)phenyl)thiazole* (**3i**). Yield: 83%, m.p. = 279–280 °C, FTIR (ATR, cm^−1^): 3097 (N–H), 1606 (C=N), 1139 (C–N), 837. ^1^H-NMR (DMSO-*d*_6_): δ = 7.32–7.37 (2H, m, benzimidazole CH), 7.60 (1H, s, thiazole CH), 7.68–7.71 (1H, m, benzimidazole CH), 7.77 (4H, m, phenyl CH), 7.80–7.81 (1H, m, benzimidazole CH), 7.91 (2H, d, *J* = 8.58 Hz, phenyl CH), 8.08 (2H, d, *J* = 8.04 Hz, phenyl CH), 8.15 (1H, s, –CH=N–), 8.61 (1H, s, benzimidazole CH), 12.37 (1H, s, –NH). ^13^C-NMR (DMSO-*d*_6_): δ = 107.16, 111.30, 120.50, 123.08, 124.08, 124.31, 124.82 (q, *J* = 270.2 Hz), 126.08 (q, *J* = 3.5 Hz), 126.54, 128.26, 126.08 (q, *J* = 37.4 Hz), 133.32, 134.05, 136.86, 138.80, 140.86, 143.63, 144.37, 149.56, 168.91. ESI-MS (*m*/*z*): [M + H]^+^: 464 (100%).

*2-(2-(4-(1H-Benzimidazol-1-yl)benzylidene)hydrazineyl)-4-(2,4-dimethylphenyl)thiazole* (**3j**). Yield: 87%, m.p. = 215–216 °C, FTIR (ATR, cm^−1^): 3097 (N–H), 1606 (C=N), 1126 (C–N), 831, 875. ^1^H-NMR (DMSO-*d*_6_): δ = 2.30 (3H, s, –CH_3_), 2.42 (3H, s, –CH_3_), 6.91 (1H, s, thiazole CH), 7.04-7.08 (2H, m, phenyl CH), 7.48–7.51 (3H, m, benzimidazole CH, phenyl CH), 7.75–7.79 (1H, m, benzimidazole CH), 7.82 (2H, d, *J* = 8.61 Hz, phenyl CH), 7.86–7.90 (1H, m, benzimidazole CH), 7.94 (2H, d, *J* = 8.70 Hz, phenyl CH), 8.15 (1H, s, –CH=N–), 9.20 (1H, s, benzimidazole CH). ^13^C-NMR (DMSO-*d_6_*): δ = 21.14, 21.53, 106.51, 112.29, 118.57, 124.83, 125.06, 125.44, 126.86, 128.15, 129.63, 131.89, 132.33, 132.57, 135.28, 135.56, 135.59, 137.22, 139.12, 140.18, 143.11, 150.98, 167.57. ESI-MS (*m*/*z*): [M + H]^+^: 424 (100%).

*2-(2-(4-(1H-Benzimidazol-1-yl)benzylidene)hydrazineyl)-4-(2,4-difluorophenyl)thiazole* (**3k**). Yield: 81%, m.p. = 274–276 °C, FTIR (ATR, cm^−1^): 3147 (N–H), 1608 (C=N), 1141 (C–N), 831, 840. ^1^H-NMR (DMSO-*d*_6_): δ = 7.19 (1H, td, *J*_1_ = 2.49 Hz, *J_2_* = 8.91 Hz, phenyl CH), 7.25 (1H, d, *J* = 2.58 Hz, phenyl CH), 7.30–7.39 (3H, m, benzimidazole CH, thiazole CH), 7.68–7.71 (1H, m, benzimidazole CH), 7.77 (2H, d, *J* = 8.70 Hz, phenyl CH), 7.80-7.86 (1H, m, benzimidazole CH), 7.91 (2H, d, *J* = 8.61 Hz, phenyl CH), 8.00–8.08 (1H, m, phenyl CH), 8.15 (1H, s, –CH=N–), 8.61 (1H, s, benzimidazole CH), 12.33 (1H, s, –NH). ^13^C-NMR (DMSO-*d_6_*): δ = 105.03 (t, *J* = 26.1 Hz), 108.48 (d, *J* = 13.5 Hz), 111.30, 112.31 (dd, *J* = 21.0 Hz−3.1 Hz), 120.50, 123.08, 124.32, 128.24, 130.85 (dd, *J* = 9.1 Hz–4.6 Hz), 133.33, 134.08, 136.88, 140.68, 140.77, 143.64, 144.00, 144.36, 144.38, 160.01 (dd, *J* = 248.2 Hz–12.6 Hz), 161.70 (dd, *J* = 246.2 Hz–11.3 Hz), 168.11. ESI-MS (*m*/*z*): [M + 2H]^2+^: 216 (100%).

*2-(2-(4-(1H-benzimidazol-1-yl)benzylidene)hydrazineyl)-4-(2,4-dichlorophenyl)thiazole* (**3l**). Yield: 84%, m.p. = 271–271 °C, FTIR (ATR, cm^−1^): 3061 (N–H), 1585 (C=N), 1139 (C–N), 825, 854. ^1^H-NMR (DMSO-*d*_6_): δ = 7.33–7.37 (2H, m, benzimidazole CH), 7.44 (1H, s, thiazole CH), 7.52 (1H, dd, *J*_1_ = 2.16 Hz, *J*_2_ *=* 8.49 Hz, phenyl CH), 7.68–7.72 (2H, m, benzimidazole CH, phenyl CH), 7.76–7.79 (3H, m, phenyl CH, benzimidazole CH), 7.91 (3H, m, phenyl CH), 8.14 (1H, s, –CH=N–), 8.61 (1H, s, benzimidazole CH). ^13^C-NMR (DMSO-*d*_6_): δ = 109.90, 111.30, 120.50, 123.08, 124.32, 127.98, 128.24, 129.34, 130.24, 132.06, 132.99, 133.33, 134.09, 136.87, 140.75, 141.47, 143.64, 144.37, 167.86. ESI-MS (*m*/*z*): [M + 2H]^2+^: 232 (100%).

### 3.2. Antifungal Activity Assays

Microbiological study was performed according to EUCAST definitive method EDef 7.1 for *Candida* species [29]. Synthesized compounds were tested for their *in vitro* growth inhibitory activity against *C. glabrata* (ATCC 90030)*, C. krusei* (ATCC 6258)*, C. parapsilosis* (ATCC 22019) and *C. albicans* (ATCC 24433). The yeasts were sustained in Roswell Park Memorial Institute (RPMI) medium, after an overnight incubation at 37 °C. The inocula of test microorganisms adjusted to match the turbidity of a MacFarland 0.5 standard tube as determined with a spectrophotometer and the final inoculum size was 0.5–2.5 × 10^5^ cfu/mL for antifungal assay. The test was performed for medium at pH = 7 and two-fold serial dilutions were applied. The last well on the microplates, which contained only the inoculated broth, was kept as control, and the last well with no growth of microorganism was recorded to represent the minimum inhibitory concentration (MIC_50_) in μg/mL. For the antifungal assays, the test compounds and reference drugs were firstly dissolved in DMSO, and further dilutions were performed to the desired concentrations of 800, 400, 200, 100, 50, 25, 12.5, 6.25, 3.125, 1.56 and 0.78 μg/mL using RPMI medium. The completed plates were incubated for 24 h, and at the end of the incubation, resazurin (20 µg/mL) was added into each well to control the growth in the wells. Final plates including microorganism strains were incubated for 2 h. MIC_50_ values were determined using microplate reader at 590 nm excitation and 560 nm emission wavelengths; MIC_50_ readings were performed twice for entire compounds. Ketoconazole and fluconazole were used as reference drugs.

### 3.3. Quantification of Ergosterol Level

Extraction of total sterols from *C. krusei* was performed as recorded by Breivik and Owades [45]. Quantification of ergosterol level in this extract was carried out in accordance with our recently described method [25].

### 3.4. Cytotoxicity Test

Cytotoxicity was experienced using the NIH/3T3 mouse embryonic fibroblast cell line (ATCC^®^ CRL-1658™, London, UK). NIH/3T3 cells were incubated according to the supplier’s references and they were seeded at 1 × 10^4^ cells into each well of 96-well plates. MTT assay was carried out as reported data [46,47,48]. Treated with the compounds at concentrations ranging from 800 µg/mL to 0.78 µg/mL. Percent inhibition was evaluated for each concentration in accordance with the following formula. Moreover, dose-response curves were plotted against compound concentrations to determine IC_50_ values [49].

### 3.5. Prediction of ADME Parameters

Physicochemical parameters of compounds (**3a**–**3l**) were analyzed by using QikProp 4.8 [37].

### 3.6. Molecular Docking Studies

A structure-based *in silico* procedure was applied to discover the binding modes of the most active compound **3c** to 14 α-sterol demethylase enzyme active sites. The crystal structures of enzyme (PDB ID: 1EA1) [40] in complex with our reference agent fluconazole, was retrieved from the Protein Data Bank server (www.pdb.org).

The structure of ligand was built using the Schrödinger Maestro [50] interface and then was submitted to the Protein Preparation Wizard protocol of the Schrödinger Suite 2016 Update 2 [51]. The ligand was prepared by the LigPrep 3.8 [52] to assign the protonation states at pH 7.4 ± 1.0 and the atom types, correctly. Bond orders were assigned and hydrogen atoms were added to the structures. The grid generation was formed using the Glide 7.1 [53] program and docking runs were performed with single precision docking mode (SP).

## 4. Conclusions

In summary, we have synthesized novel benzimidazole-thiazole derivatives showing significant anticandidal effects. Additionally, antifungal activity studies, quantification of ergosterol level, preliminary toxicological screening, ADME prediction and docking evaluations of obtained compounds were undertaken in the current study. Activity studies showed that the compound **3c** was the most active compound in the series, with a MIC_50_ value of 1.56 µg/mL against *Candida* strains. Toxicological and ADME studies enhanced the biological importance of this compound. Docking evaluations demonstrated the binding modes of this compound to enzyme active site. We presume that in further studies all these findings may help medicinal chemists to discover more promising anticandidal compounds.

## Figures and Tables

**Table:** 

Compounds	R_1_	R_2_
**3a**	–H	–H
**3b**	–H	–CH_3_
**3c**	–H	–NO_2_
**3d**	–H	–CN
**3e**	–H	–OCH_3_
**3f**	–H	–F
**3g**	–H	–Cl
**3h**	–H	–Br
**3i**	–H	–CF_3_
**3j**	–CH_3_	–CH_3_
**3k**	–F	–F
**3l**	–Cl	–Cl

**Table 1:** MIC_50_ (µg/mL) values of compounds (**3a**–**3l**).

Compounds	*C. albicans*	*C. glabrata*	*C. krusei*	*C. parapsilopsis*
**3a**	100	25	100	100
**3b**	100	25	100	100
**3c**	1.56	1.56	1.56	1.56
**3d**	3.12	1.56	1.56	3.12
**3e**	6.25	100	100	100
**3f**	6.25	25	6.25	6.25
**3g**	6.25	12.5	6.25	6.25
**3h**	100	12.5	100	100
**3i**	100	100	100	100
**3j**	50	100	100	100
**3k**	6.25	6.25	12.5	6.25
**3l**	6.25	12.5	6.25	6.25
Ketoconazole	0.78	1.56	1.56	1.56
Fluconazole	0.78	1.56	1.56	0.78

**Table 2:** Inhibition potency (%) of compounds **3c** and **3d** on ergosterol biosynthesis of *C. albicans*.

Compounds	Concentrations (µg/mL)		
	0.78	1.56	3.12
**3c**	56.83 ± 2.96	65.81 ± 3.88	79.14 ± 4.29
**3d**	48.25 ± 3.17	58.77 ± 4.03	66.58 ± 3.27
Ketoconazole	60.99 ± 2.94	73.12 ± 4.16	84.56 ± 3.01
Fluconazole	61.74 ± 1.70	70.12 ± 3.22	82.13 ± 4.45

**Table 3:** Cytotoxic activity of the compounds **3c** and **3d** against NIH/3T3 Cell Line.

Compounds	IC_50_ (µg/mL)	*R*^2^
**3c**	>500	0.9754
**3d**	>500	0.9839

**Table 4:** Calculated ADME parameters of compounds **3a**–**3l**.

Compounds	MW	RB	MV	DHB	AHB	PSA	log P	VRT	VRF
**3a**	395.48	4	1263.60	1	5.5	51.98	5.33	1	1
**3b**	409.51	4	1322.54	1	5.5	51.98	5.63	1	1
**3c**	440.48	5	1346.00	1	6.5	100.64	4.62	0	1
**3d**	420.49	5	1330.30	1	7	77.77	4.56	0	1
**3e**	425.51	5	1331.38	1	6.25	60.46	5.37	1	1
**3f**	413.47	4	1279.71	1	5.5	51.98	5.56	1	1
**3g**	429.93	4	1307.72	1	5.5	51.98	5.82	1	1
**3h**	474.38	4	1316.63	1	5.5	51.98	5.90	1	1
**3i**	463.48	4	1360.43	1	5.5	51.98	6.31	1	1
**3j**	423.53	4	1346.88	1	5.5	47.09	5.80	1	1
**3k**	431.46	4	1289.37	1	5.5	50.54	5.72	1	1
**3l**	464.37	4	1316.53	1	5.5	48.57	6.00	1	1
Ketoconazole	530.45	5	1511.39	0	6.75	55.75	4.91	1	0
Fluconazole	306.27	6	883.50	1	6.75	72.55	3.55	0	0

MW: Molecular weight; RB: Number of rotatable bonds (recommended value: 0–15); MV: Total solvent-accessible volume in cubic angstroms using a probe with a 1.4 Å radius (recommended value: 500–2000); DHB: Estimated number of hydrogen bonds that would be donated by the solute to water molecules in an aqueous solution (recommended value: 0–6); AHB: Estimated number of hydrogen bonds that would be accepted by the solute from water molecules in an aqueous solution (recommended value: 2–20); PSA: Van der Waals surface area of polar nitrogen and oxygen atoms and carbonyl carbon atoms (recommended value: 7–200); log P: Predicted octanol/water partition coefficient (recommended value: −2–6.5); VRF: Number of violations of Lipinski’s rule of five [38]. The rules are: mol_MW < 500, QPlogPo/w < 5, donorHB ≤ 5, accptHB ≤ 10. Compounds that satisfy these rules are considered druglike. (The “five” refers to the limits, which are multiples of 5.) (maximum is 4); VRT: Number of violations of Jorgensen’s rule of three [39]. The three rules are: QPlogS > −5.7, QP PCaco > 22 nm/s, # Primary Metabolites < 7. Compounds with fewer (and preferably no) violations of these rules are more likely to be orally available (maximum is 3).

## Supplementary Materials

Supplementary materials are available online.
