# Supplementary material for: Synthesis and Anticandidal Activity Evaluation of New Benzimidazole-Thiazole Derivatives
Source: Molecules. 2017 Nov 23;22(12):2051. doi: 10.3390/molecules22122051 (PMC6149685; doi:10.3390/molecules22122051)
Supplement: Supplementary file 1 [file molecules-22-02051-s001.pdf]

Synthesis and Anticandidal Activity Evaluation of New Benzimidazole-thiazole Derivatives

Zafer Asım Kaplancıklı [1,*], Serkan Levent [1,2], Derya Osmaniye [1,2], Begüm Nurpelin Sağlık [1,2], Ulviye Acar Çevik [1,2], Betül Kaya Çavuşoğlu [1], Yusuf Özkay [1,2] and Sinem Ilgın [3]

1 Department of Pharmaceutical Chemistry, Faculty of Pharmacy, Anadolu Universty, Eskişehir, Turkey
2 Doping and Narcotic Compounds Analysis Laboratory, Faculty of Pharmacy, Anadolu Universty, Eskişehir, Turkey
3 Department of Pharmaceutical Toxicology, Faculty of Pharmacy, Anadolu Universty, Eskişehir, Turkey
* Correspondence: zakaplan@anadolu.edu.tr; Tel.: +90-222-335-0580/1180

DOPNALAB

	Item	Value
	Acquired Date&Time	11.10.2017 11:18:18
	Acquired by	System Administrator
	Filename	C:\Users\dopnalab\Desktop\derya\BZ serisi\BZ-31.ispd
	Spectrum name	BZ-31
	Sample name	BZ-3
	Sample ID	
	Option	
	Comment	
	No. of Scans	10
	Resolution	4 [cm-1]
	Apodization	Happ-Genzel

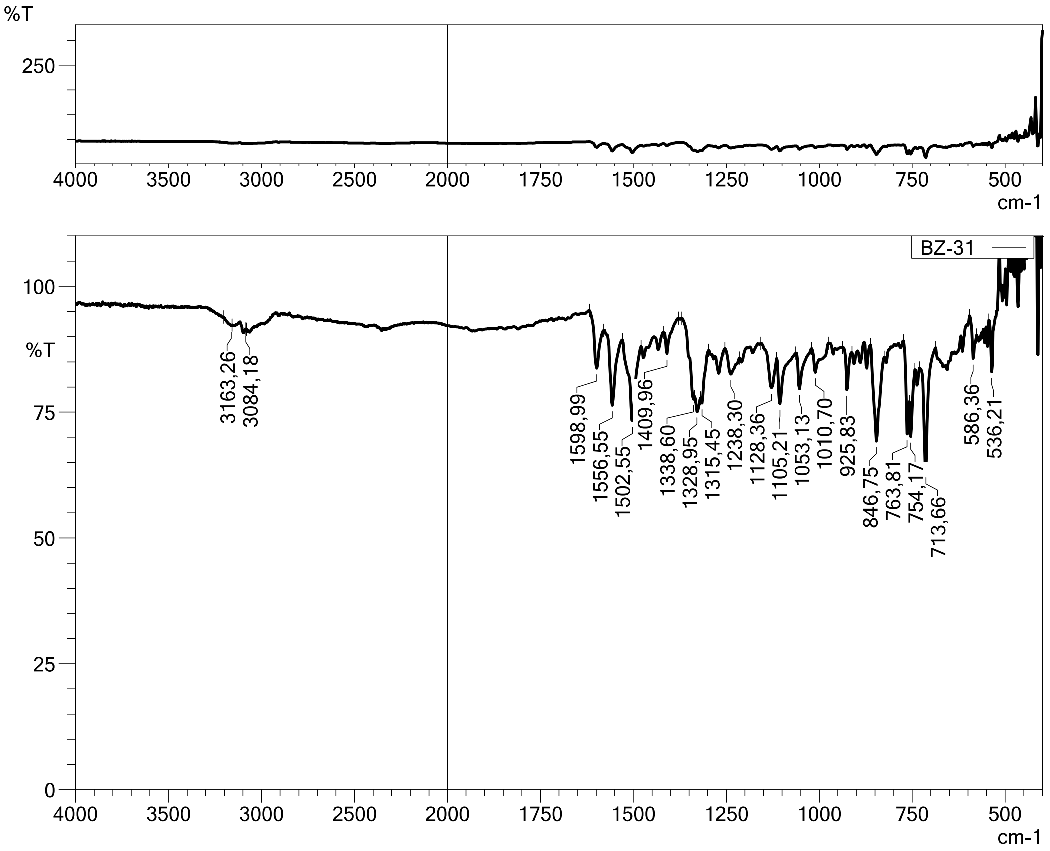

Figure S1. Compound **3c** IR spectrum

LCMSMS ANALİZ RAPORU

Sample Name	: BZ-3		
Sample ID	:		
Data Filename	: BZ-3_013.lcd		
Method Filename	: Muratsentez.lcm		
Batch Filename	: sentez1.lcb		
Vial #	: 1-3		
Injection Volume	: 1 uL	Sample Type	: Unknown
Date Acquired	: 27.10.2017 15:14:36	Acquired by	: System Administrator
Date Processed	: 27.10.2017 15:16:39	Processed by	: System Administrator

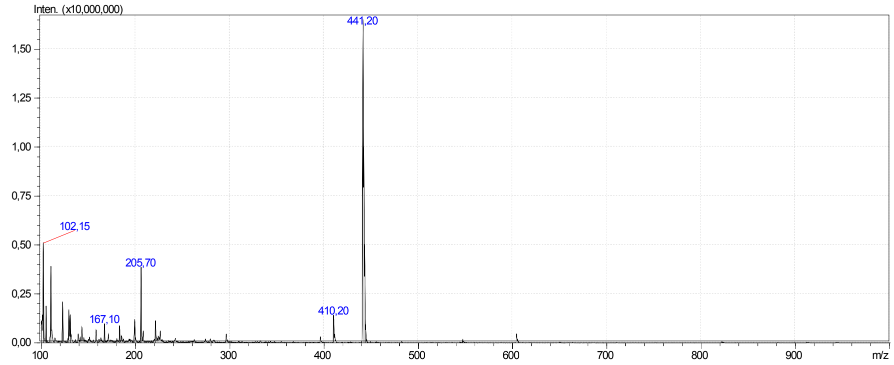

[MS Spectrum]

# of Peaks	17
Raw Spectrum	[0,034->0,609],(scan:[3->37])
Background	No Background Spectrum
Base Peak	m/z 441,20 (Inten : 16.534.486)

m/z	Absolute Intensity	Relative Intensity
183,10	881057	5,33
199,00	1214039	7,34
205,70	3859284	23,34
221,20	1131750	6,84
410,20	1409243	8,52
441,20	16534486	100,00
442,20	10021220	60,61
443,20	5039385	30,48
444,20	936814	5,67

Figure S2. Compound **3c** mass spectrum

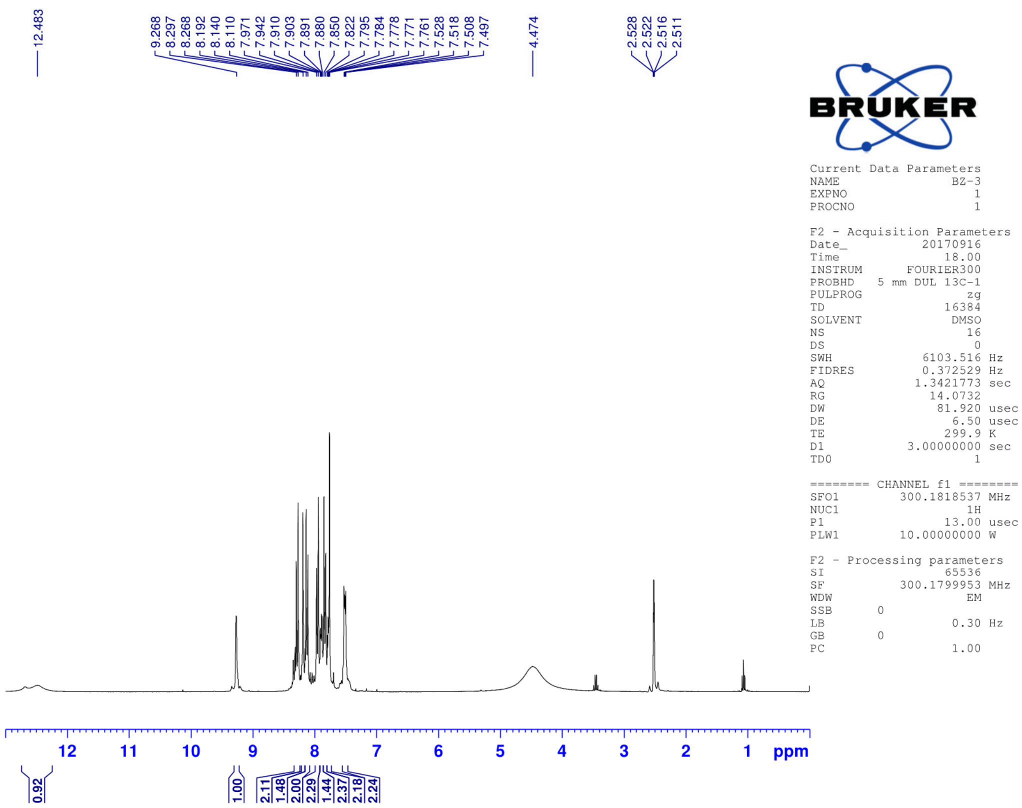

Figure S3. Compound **3c** ^{1}H-NMR spectrum

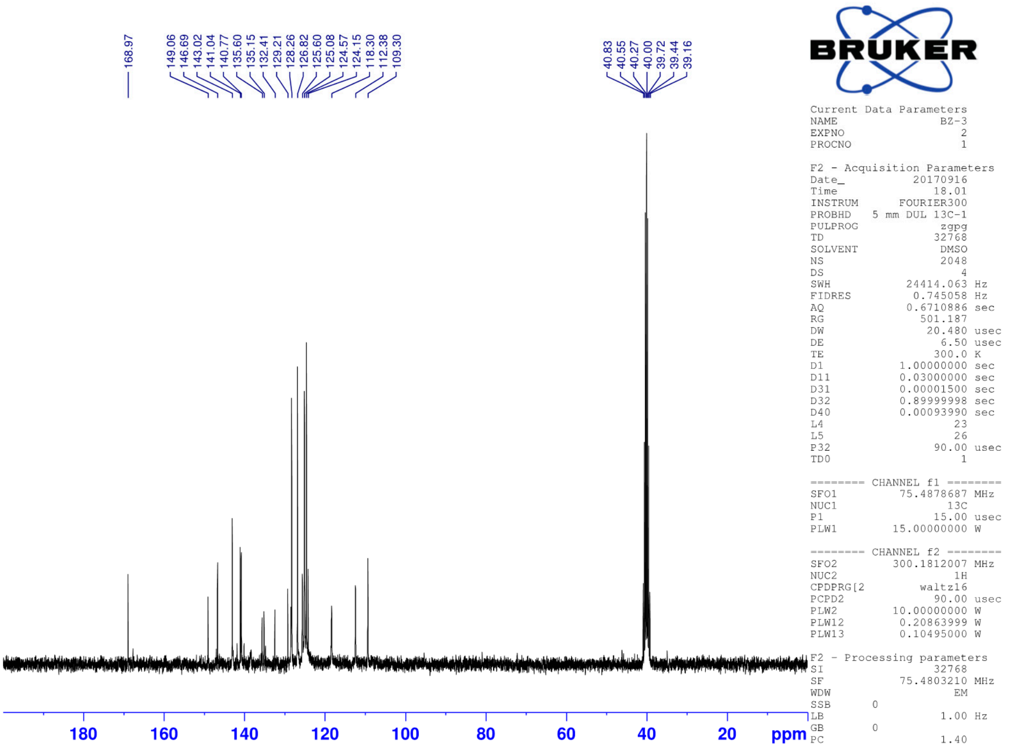

Figure S4. Compound **3c** ^{13}C-NMR spectrum

DOPNALAB

	Item	Value
	Acquired Date&Time	11.10.2017 11:20:35
	Acquired by	System Administrator
	Filename	C:\Users\dopnalab\Desktop\derya\BZ serisi\BZ-41.ispd
	Spectrum name	BZ-41
	Sample name	BZ-4
	Sample ID	
	Option	
	Comment	
	No. of Scans	10
	Resolution	4 [cm-1]
	Apodization	Happ-Genzel

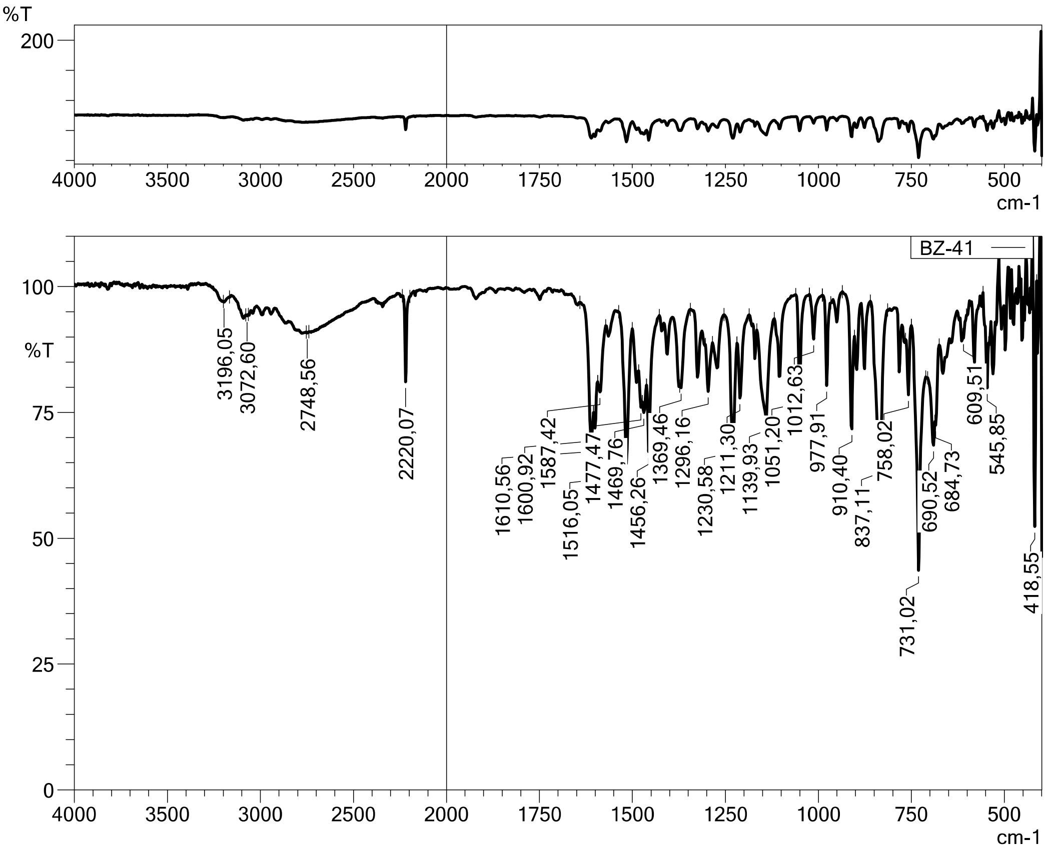

Figure S5. Compound **3d** IR spectrum

LCMSMS ANALİZ RAPORU

Sample Name	: BZ-4		
Sample ID	:		
Data Filename	: BZ-4_014.lcd		
Method Filename	: Muratsentez.lcm		
Batch Filename	: sentez1.lcb		
Vial #	: 1-5		
Injection Volume	: 1 uL	Sample Type	: Unknown
Date Acquired	: 27.10.2017 15:17:13	Acquired by	: System Administrator
Date Processed	: 27.10.2017 15:19:17	Processed by	: System Administrator

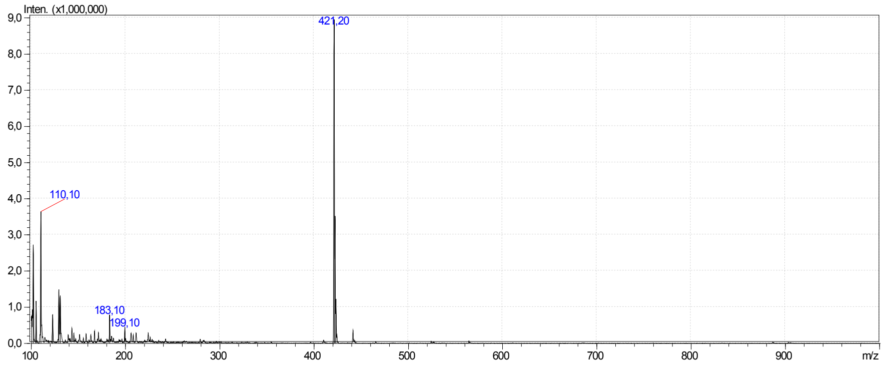

[MS Spectrum]

# of Peaks 12

Raw Spectrum [0,101->0,711],(scan:[7->43])

Background No Background Spectrum

Base Peak m/z 421,20 (Inten : 8.962.321)

m/z	Absolute Intensity	Relative Intensity
122,65	795496	8,88
129,20	1489047	16,61
130,60	1316282	14,69
183,10	794270	8,86
199,10	448404	5,00
421,20	8962321	100,00
422,20	3519323	39,27
423,20	1225991	13,68

Figure S6. Compound **3d** mass spectrum

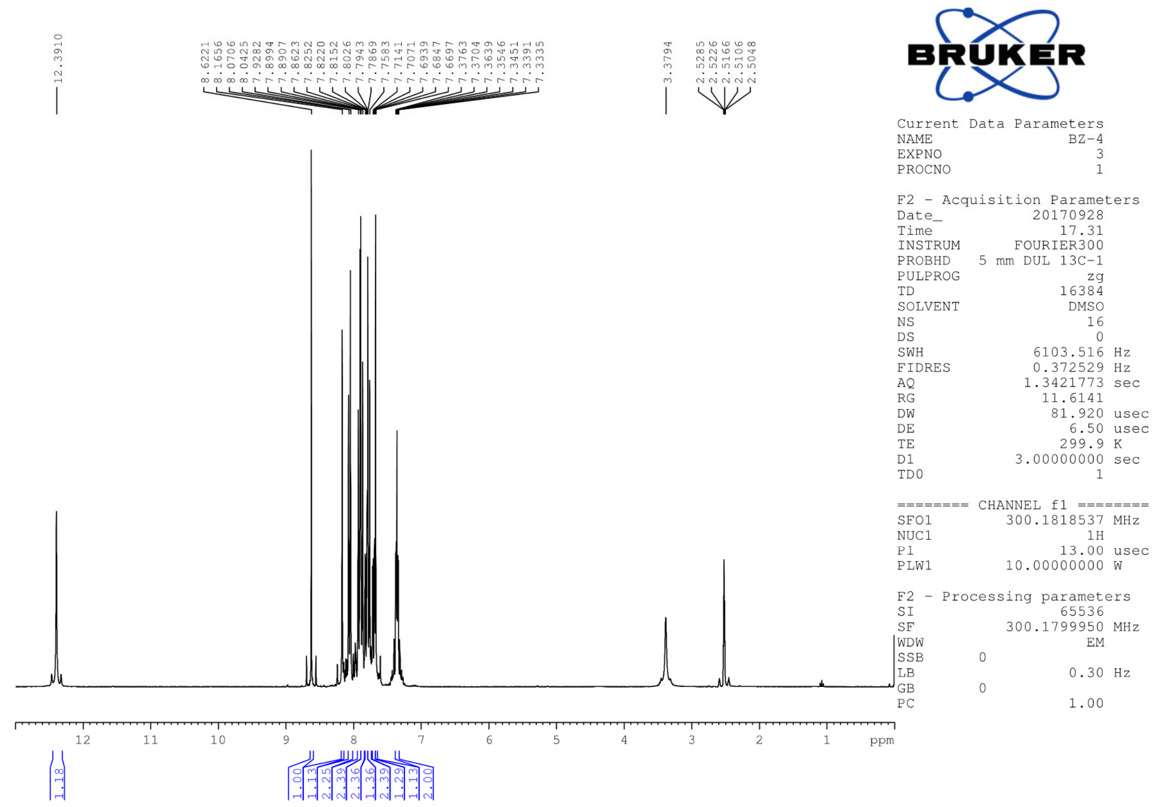

Figure S7. Compound **3d** ^{1}H-NMR spectrum

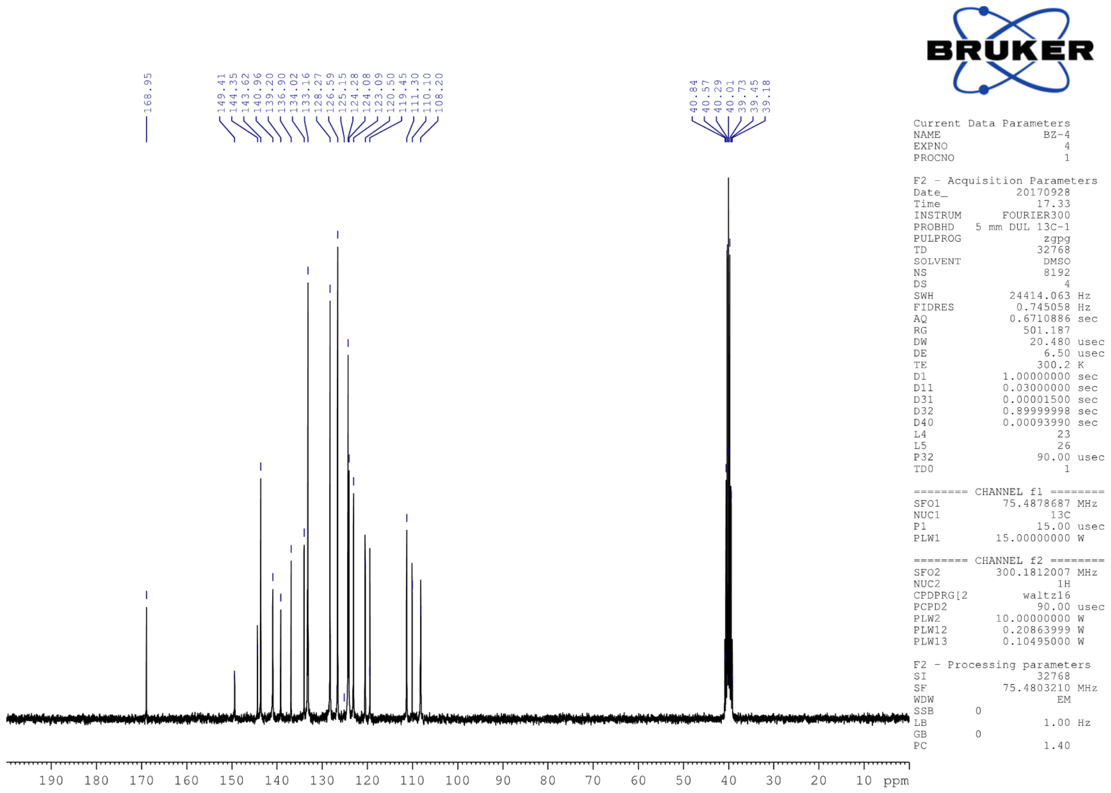

Figure S8. Compound **3d** ^{13}C-NMR spectrum
